# Vitamin C, Aging and Alzheimer’s Disease

**DOI:** 10.3390/nu9070670

**Published:** 2017-06-27

**Authors:** Fiammetta Monacelli, Erica Acquarone, Chiara Giannotti, Roberta Borghi, Alessio Nencioni

**Affiliations:** Section of Geriatrics, Department of Internal Medicine and Medical Specialties (DIMI), University of Genoa, Genoa 16132, Italy; erica88im@hotmail.com (E.A.); chiara.giannotti86@gmail.com (C.G.); robertaborghi@yahoo.it (R.B.); Alessio.Nencioni@unige.it (A.N.)

**Keywords:** ascorbic acid, aging process, Alzheimer’s disease

## Abstract

Accumulating evidence in mice models of accelerated senescence indicates a rescuing role of ascorbic acid in premature aging. Supplementation of ascorbic acid appeared to halt cell growth, oxidative stress, telomere attrition, disorganization of chromatin, and excessive secretion of inflammatory factors, and extend lifespan. Interestingly, ascorbic acid (AA) was also found to positively modulate inflamm-aging and immunosenescence, two hallmarks of biological aging. Moreover, ascorbic acid has been shown to epigenetically regulate genome integrity and stability, indicating a key role of targeted nutrition in healthy aging. Growing in vivo evidence supports the role of ascorbic acid in ameliorating factors linked to Alzheimer’s disease (AD) pathogenesis, although evidence in humans yielded equivocal results. The neuroprotective role of ascorbic acid not only relies on the general free radical trapping, but also on the suppression of pro-inflammatory genes, mitigating neuroinflammation, on the chelation of iron, copper, and zinc, and on the suppression of amyloid-beta peptide (Aβ) fibrillogenesis. Epidemiological evidence linking diet, one of the most important modifiable lifestyle factors, and risk of Alzheimer's disease is rapidly increasing. Thus, dietary interventions, as a way to epigenetically modulate the human genome, may play a role in the prevention of AD. The present review is aimed at providing an up to date overview of the main biological mechanisms that are associated with ascorbic acid supplementation/bioavailability in the process of aging and Alzheimer’s disease. In addition, we will address new fields of research and future directions.

## 1. Ascorbic Acid and Its Relevance to the Aging Process

Due to the aging population and the increased life-expectancy [1] in developed and developing countries, a growing area of interest concerns the understanding of the mechanisms that regulate aging and that differentiate successful aging from pathological aging. Prolonged exposure to antigens throughout life produces a progressive modification of the individual homeostasis [2].

The free radical theory of aging allows an explanation of the molecular mechanisms underlying the aging process, at least partially, and the pathogenesis of age-related diseases, such as atherosclerosis, cardiovascular diseases, dementia, diabetes, and osteoporosis [3,4]. From a biological perspective, the process of aging is characterized by immunosenescence: this may be defined as the reduced ability to respond to foreign antigens and to tolerate self-antigens, leading to increased susceptibility to infections, cancer, and autoimmune diseases [5]. The most accredited molecular mechanisms for immunosenescence include redox-mediated and mitochondria-dependent oxidative pathways. High levels of free radicals and peroxidation products of lipid membranes, such as malondialdehyde (MDA), are able to modulate the activation of nuclear transcription factors, associated with cell aging and longevity, such as tumor protein p53, transcriptional protein AP-1 and nuclear factor kappa-light-chain-enhancer of activated B cells (NF-kB). In addition, these same mechanisms are responsible for altered T cell expression and the altered phenotype of immunological subpopulations [6].

Oxidative stress is increasingly considered to be the major epigenetic factor for aging and also plays an important role in inducing the low-grade inflammation, as the two processes are strictly intertwined. This pro-inflammatory phenotype, defined ‘inflamm-aging’, is characterized by an increased expression of genes related to inflammation and to the immune response. Indeed, it is known that the increased serum levels of C reactive protein (CRP) and pro-inflammatory cytokines, such as interleukin-6 (IL-6) and tumor necrosis factor alpha (TNF-α) induce the activation of NF-KB mediated superoxide production in mitochondria, promoting the release of oxygen reactive species (ROS) [2,6].

Inflamm-aging is also associated with decreased nitric oxide (NO) bioavailability in the endothelial layer, which induces endothelial dysfunction. Inflamm-aging may be considered a biological background for both the aging process and the pathophysiological process of frailty in humans [7].

The endogenous antioxidant enzymatic defense system superoxide dismutase (SOD), catalase and glutathione peroxidase (GSH) counteracts oxidative exogenous agents from diet and may undergo substantial decrease related to the aging process [8]. Accumulating evidence indicates that nutrition is a key relevant factor for inflamm-aging and an important modulator of the aging process as well [9,10,11].

Ascorbic acid (AA) is a powerful first-line antioxidant that mediates several beneficial effects on redox oxidative pathways and mitochondrial pathways on the immune system, on inflamm-aging, on endothelial integrity, and on lipoprotein metabolism [12,13]. AA, a lactone with six carbon atoms, is synthesized from glucose in the liver of many species of mammals. Humans have evolutionarily lost the gulonolactone oxidase enzyme, essential for the synthesis of 2-keto-l-gulonolactone, its direct precursor. As a result, people absorb AA exclusively from the diet. AA enters the cells through the sodium-dependent vitamin C transporters SVCT1 and SVCT2, a process favored by the electrochemical sodium gradient: due to its high capacity and low affinity, SVCT1 assures the intestinal and renal absorption and reabsorption [14].

All of the physiological and biochemical actions of AA are due to its ability to donate electrons (as a reducing agent). AA undergoes two consecutive and reversible oxidations: from the first electron loss, it generates an intermediate product, the ascorbate free radical (AFR), which is converted into dehydroascorbate (DHA) after the loss of the second electron. At physiological concentrations, AA is a powerful antioxidant and scavenger of free radicals in plasma and different tissues, including the central nervous system (CNS) [15,16].

AA is also implicated in the endothelial integrity associated with NO bioavailability [17]. The molecular mechanisms that induce endothelial dysfunction affect the enzyme NO synthase (eNOS) by impairing Gi- dependent signaling, decreasing mRNA stability for eNOS, and blocking eNOS translocation from the plasma membrane to the Golgi membranes. The reduced bioavailability of its substrate (l-arginine) or its co-factor (tetrahydrobiopterin BH4) was also observed [18]. Low levels of BH4 compromise eNOS function by promoting the transfer of electrons to oxygen molecules instead of l-arginine: in turn, eNOS produces a superoxide anion instead of generating NO [19].

## 2. Ascorbic Acid, Epigenetic Modulation and Nutrigenomics

In the last decades, the understanding of AA properties has undergone a major revolution, ranging from a simple antioxidant to a micronutrient, capable of epigenetic regulation [20,21].

Recent advances in epigenetics have identified a series of di-oxygenases Fe^2+^ and 2 oxoglutarate (2OG-dependent) enzymes that catalyze the epigenetic modifications of DNA and histones. Some of these enzymes require ascorbate to maintain their catalytic activity. Therefore, the availability of ascorbate might affect the epigenome, with a potential impact on health and age-related diseases. Methylation in the C5 position of cytosine (5-methylcytosine, 5mC) is the most important and well-studied epigenetic mark of mammalian DNA, which plays an essential role in the transcriptional and in the maintenance of genome stability.

DNA methyltransferase (DNMTs) is responsible for the transfer of a methyl group from the universal methyl donor, *S*-adenosyl-l-methionine (SAM), to the 5-position of cytosine residues in DNA. The presence of an unusual nucleotide, 5-hydroxymethylcytosine (5hmC) in mammalian DNA has been reported. Although 5hmC represents less than 1% of total nucleotides, high levels have been observed in the cerebellar Purkinje cells and in the granule neurons, suggesting a potential role for neuronal functions in epigenetic regulation. This nucleotide (5hmC) is formed by the activity of a group of enzymes, ten-eleven translocation methylcytosine dioxygenas (TET: TET1, TET2, TET3), that catalyze the ten-eleven translocation and oxidize 5mC to generate 5hmC. TET enzymes were shown to further oxidize 5hmC into 5-formylcytosine (5fC) and 5-carboxylcytosine (5caC). Ascorbic acid is known to increase 5hmC production in a TET-dependent way, probably by reactivating the catalytic site of TET enzyme, reducing Fe^3+^ to Fe^2+^. Namely, AA induced a significant demethylation of 5-methylcytosine (5mC) to 5-hydroxymethyl cytosine (5hmC) [22,23].

Variation in ascorbate bioavailability can influence the demethylation of DNA and histones: in addition, ascorbate deficiency can present at different stages of aging and could be involved in the development of different age-related diseases. In particular, if additional ascorbate is not provided by supplementation or improved uptake, there would be progressive AA decline in the brain, which might be associated with neurodegeneration. So far, there is inconsistent data on epigenetic modifications in the human brain. Further studies could unravel the potential impact of age-related ascorbate decline on the epigenome and on neurodegeneration.

So far, AA seems to increasingly have beneficial effects on the aging processes and on the prevention of age-related diseases as atherosclerosis, cardiovascular diseases, cancer, and neurodegenerative diseases.

Nutrigenomics is a young area of research, but meaningful studies indicated a role for AA on gene expression. A previous study found that, although no diet-gene interactions were observed, genetic variation of SVCT1 can influence serum ascorbic acid concentrations. Moreover, both AA transporter genotypes modify the strength of the correlation between dietary AA and serum levels [24].

Recently, genetic variations of haptoglobin, polymorphisms in the transporters of AA, and deleted polymorphisms of glutathione-*S*-transferase have provided genetic information regarding possible relative AA levels [25,26,27].

**Figure 1:**
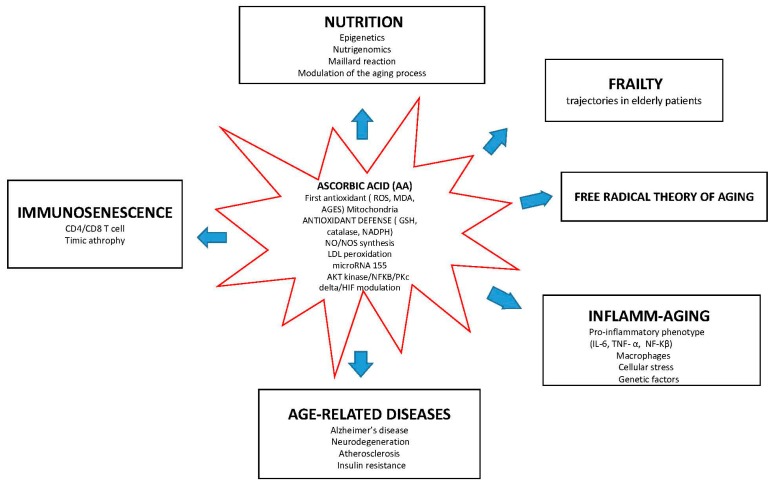
AA is at the crossroads of biological aging, intercepting immunosenescence, inflamm-aging, and oxidative stress (free radical theory of aging), with a potential role in the onset of age-related diseases and frailty trajectories.

Intriguingly, AA functions at the interface of different molecular pathways associated with aging, as illustrated by Figure 1.

## 3. Ascorbic Acid and the Aging Process: In Vitro Models

Several lines of research have increasingly accredited the role of AA in the aging process. The in vitro and in vivo evidence is reported herein and illustrated in Table 1.

A mouse model of senescence showed that AA promotes proliferation of bone marrow mesenchymal cells derived from aging mice. The senescence-accelerated mouse prone 6 (SAMP6) mice and senescence-accelerated mouse resistant 1 (SAMR1) mice were used as the test group and the control group, respectively. Bone marrow mesenchymal stem cells (BMMSCs) derived from SAMP6 mice were treated with increasing concentrations of AA [44]. The treatment significantly improved the BMMSCs proliferation in a dose-dependent manner by increasing telomerase activity and TERT expression. The AA concentration of 100 µg/mL induced the strongest effect in promoting the proliferation of BMMSCs in SAMP6 mice, while at a concentration of 1.000 mg/mL, AA suppressed the cell growth. AA can promote the proliferation of BMMSCs from aging mice, possibly by increasing the cellular telomerase activity. 

Interestingly, it is known that bone marrow (BM) plays a key role in immunological memory and surveillance, through inflamm-aging. Overexpression of IL-15 and IL-6 was stimulated by IFN-y and correlated with ROS. The plasma-cell survival factor a proliferation- inducing ligand (APRIL) was also reduced. AA was effective in counteracting inflammatory- and oxidative stress-related changes in the aging bone marrow, improving immunological memory in old age. This study is of key relevance in assessing the protective role for AA in immunosenescence [35].

The positive effects of AA on premature cellular events was confirmed by treatment with AA on Werner’s syndrome protein (WRN-deficient) human mesenchymal stem cells (MSCs) [33]. In this model, the analysis of mRNA levels showed that AA altered gene expression was involved in chromatin condensation, the regulation of the cell cycle, and DNA replication and repair. AA promoted heterochromatin remodeling to a younger state (as demonstrated from the upregulation of heterochromatin Protein 1 (HP1α) markers and histone H3K9me3 by Western blotting). AA slowed down cellular senescence in mesenchymal WRN-deficient cells (as demonstrated by the SA-β-gal staining) restoring mesenchymal stem cells’ vitality and proliferative potential. AA repressed telomere shortening, decreased the production of pro-inflammatory cytokines, such as IL-6 and IL-8, downregulated the expression of markers of aging, such as cyclin-dependent kinase inhibitor 2A, multiple tumor suppressor 1 p16Ink4a and zinc finger transcriptional GATA4, and repressed the SASP (elevated senescence-associated secretory phenotype). AA was effective at alleviating aging defects by reducing cell cycle regulation, telomere attrition, ROS burst, and nuclear laminin disorganization.

AA was also reported to stimulate/inhibit the differentiation of mesoderm-derived embryonic stem cells (ES) through the involvement of p38 mitogen activated protein kinase/cAMP response element binding (CREB) nuclear transcription factor activation (p38 MAPK/CREB pathway) and increased expression of the SVCT2 transporter. More precisely, AA was found to promote ES differentiation by regulating chromatin domain overlapping. These in vitro models have important implications for the aging process. The effect of AA in the context of body weight could be at least partially related to stem cell differentiation towards myogeneis and osteogenesis, inhibiting adipogenesis. Since aging is associated with sarcopenia and defects in body weight, the current observations suggest that AA-mediated stem cell effects could play a role in the aging process [36,37].

Furthermore, the same results were replicated in human bone marrow mesenchymal stromal cells (hBM-MSCs) undergoing replicative senescence to investigate the relationship between ROS levels and stem cell potential differentiation after AA treatment. Interestingly, AA supplementation eliminated ROS excess and restored the endogenous antioxidant enzymatic activity (catalase, SOD) by influencing phosphorylated fox head box O protein 1 (p-FOXO) and p53. Moreover, differentiation into adipocytes and osteocytes was significantly increased [38].

Thus, AA seems to be implicated in the regulation and differentiation of stem cells. Current knowledge on MSC cell surface biomarkers and molecular mechanisms of MSC differentiation emphasizes the role of Wnt/β-catenin signaling, the Notch signaling pathway, bone morphogenesis proteins, various growth factors, and oncogene and immunosuppressive activities of MSCs. Therefore, further investigations are needed to establish a role for AA in such targeting regulation of cell differentiation, which may have important clinical implications for the prevention of age-related disease [45].

### Ascorbic Acid and the Aging Process: In Vivo Evidence

Murine models of Werner’s syndrome (WS) (Wrn Δhel/Δhel mutants) exhibit many phenotypic characteristics similar to accelerated human aging. The supplementation with AA for nine months was found to reduce oxidative stress in hepatocytes and cardiomyocytes, and decrease hypertriglyceridemia and hyperglycemia. A significant improvement of the metabolic profile, including insulin resistance and the body fat in WrnΔhel/Δhel mutant mice was also observed.

Similarly, other Werner syndrome-like in vivo models have confirmed that AA supplementation rescued the shorter lifespan, reversing age-related abnormalities in adipose tissue, the liver, and genome integrity. In the metabolic profile, inflammatory status was improved and, at a molecular level, the normalization of the phosphorylation of AKT kinase, transcriptional levels of NF-kappa B, protein kinase delta (PKC delta), peroxisome proliferator-activated receptor alpha (PPAR-alpha) and hypoxia-inducible factor-1 alpha (HIF1-alpha), were observed [45].

A further study of WrnΔhel/Δhel mutant mouse models showed that the pro-oxidant and inflammatory state produced a premature defenestration of sinusoidal endothelium in liver tissue with consequent hepatic dysfunction and impaired hepatic lipoprotein metabolism. Long-term treatment with AA restored physiological levels of GSH and the fenestrated sinusoidal endothelium by quenching oxidative stress. It is noteworthy that in healthy mice, the beneficial effects of AA on health and lifespan were not significant and the supplementation significantly reduced the oxidative damage only in Wrn∆hel/∆hel mouse liver [30,34].

AA had a positive impact on the cardiometabolic and inflammatory profiles of mice lacking the functional Werner syndrome protein helicase. AA reversed changes in the expression levels of plasminogen activator-1 (PAI-1) and improved, at a transcriptional level, fatty acid degradation. In addition, AA increased glutathione metabolism and reversed the oxidative stress. This study suggested that AA could be a potential cardiometabolic biomarker in patients with WS [39].

*Caenorhabdilis elegans* worm models, with a non-functional wrn-1 DNA helicase ortholog, exhibited a shorter lifespan. The supplementation with AA increased lifespan in the mutant strain, compared with the wild-type strain possibly by altering the expression of genes regulating the metabolism of lipids, ketones, organic acids, and carboxylic acids. AA modified the expression of genes involved in locomotion and development of the anatomical structure. Conversely, in the wild-type worms, AA only influenced the biological process of proteolysis [32].

Knockout (Gulo−/−) mice represent an interesting in vivo model that mimics human physiology, lacking the gulonolactone oxidase (Gulo) gene. Accumulating evidence from this in vivo model yielded additional information on the role of ascorbate in aging. Knockout (Gulo−/−) mice developed high oxidative stress, sensorimotor deficits, and behavior abnormalities. The lifespan of Gulo−/− mice appeared to inversely correlate with the phosphorylation levels of IRE1α and IF2α, in response to endoplasmic reticulum stress. In this model, AA supplementation reduced phosphorylated IRE1α, implicating its protective effect on endoplasmic reticulum stress and extended lifespan. In addition, in the same in vivo model, AA supplementation was shown to improve T cell-mediated acute response after liver injury, suggesting a modulation of the immune system [31].

Uchio et al. demonstrated the influence of long-term high-dose AA intake on the number and function of immune cells in SMP30KO mice. The total counts of leucocytes, lymphocytes, granulocytes, and monocytes in the peripheral blood, as well as the number of splenocytes and thymocytes, were all significantly higher in the treated group. In addition, the number of naive T cells in peripheral blood lymphocytes, the number of memory T-cell populations in splenocytes, and the number of clusters of differentiated CD4+ and CD8+ T cells in thymocytes were all remarkably elevated. High dietary AA intake was associated with the improvement of age-related thymic atrophy. The study indicated a role for AA in immunosenescence by targeting CD4+ and CD8+ cells. Further, AA was found to modulate immune cell surveillance in SMP30 knockout mice [40].

In line with these data, Sato and colleagues suggested that AA plays an important role in preventing protein oxidation in the liver of SM30/gluconolactonase knockout mice, with potential implications in overall health and aging [41].

## 4. Ascorbic Acid and the Aging Process: Oxidative Stress and Antioxidant Defense

Oxidative stress is considered noxious for lifespan and AA, and, as a first line antioxidant, has been thought to potentially increase longevity. These notions have recently been challenged by findings in model organisms that show beneficial effects on lifespan of increased ROS generation produced by mutations or pro-oxidant treatments [46,47]. Such a relationship would arise from a combination of beneficial effects from a moderate increase in ROS levels and their dose-dependent toxicity. Intriguingly, the small elevation of ROS levels that increase lifespan seems not to be stressful, nor do they induce an increased resistance to oxidative stress. In particular, in a *Caenorhabdilis elegans* model, [48] AA displayed an inverted U-shaped dose–response relationship between ROS levels and lifespan; both high and low levels of ROS were detrimental for longevity. This evidence further complicated the role of AA in aging. The fact that both antioxidant and pro-oxidant treatments reveal such a different behavior suggests that temporal administration of AA is of key relevance to obtain beneficial effects. Moreover, ROS levels still need to be optimized for lifespan in different cells, and the net balance between antioxidant and oxidant defense and their concentrations plays a relevant role in the aging process. 

From a clinical perspective, AA functions at a true interface between aging, life span and age-related diseases. It is able to modulate telomeres activity, bioenergetics, DNA repair and oxidative stress, indicating a nutrigenomic role in the process of aging as well [49]. 

During aging, the antioxidant capacity of AA is finely regulated by the redox balance of DHA/ascorbate and the ability of the endogenous antioxidant enzymatic defense system (glutathione and nicotinamide adenine dinucleotide phosphate; NADPH) to recycle DHA back to AA. An increased ratio of DHA/AA becomes an indicator of a pro-oxidative ability of AA to mediate biological processes and may play a role in age-related diseases, intercepting different aging trajectories. 

Similarly, cellular antioxidant enzymatic capacity declines during the aging process and oxidation of glutathione and NADPH may also explain different results in studies targeting aging and disease prevention. Recently, it has been shown that elderly people with lower peripheral antioxidant parameters, including AA and a decreased antioxidant capacity, are more prone to clinical vulnerability, disability, frailty and higher mortality over a 5-year follow up [50]. 

Conversely, two studies in healthy elderly subjects showed that daily intake of star fruit juice (Averrhoa Carambola, a fruit with high content of AA) acted as a scavenger of free radicals, and maintained low levels of lipoperoxidative stress (MDA), restoring GSH levels. The associated AA antioxidant capacity also mediated anti-inflammatory effects by the reduction of pro-inflammatory cytokine secretion, especially TNF-alpha and interleukin-23 (IL-23) excluding interleukin-2 (IL-2) [51,52]. 

Kim et al. investigated the effects of high-dose AA supplementation (1250 mg daily) in humans. After eight weeks, the analysis of serum lipoproteins showed a reduction of advanced glycation end products (AGEs). The anti-glycoxidative effect was significantly higher, especially in non-smoking men, and was associated with net improvement of plasma HDL levels. The quantitative analysis of the LDL fractions also showed an improvement of LDL lipid composition. Therefore, the supplementation with AA could exert protective effects against atherosclerosis and related systemic inflammation by reducing the oxidized LDL and macrophage phagocytosis, with reduced conversion to foam cells [42].

Interestingly, this study demonstrated that AA induced changes in gene expression of some microRNAs that negatively regulate target genes’ post-transcriptional expression. After AA consumption, miR155 levels decreased by 90%, suggesting that high doses of AA may significantly modulate miRNA levels and the anti-inflammatory response [42]. Thus, AA may be considered an epigenetic key to personalized nutrition.

## 5. Evidence for Ascorbic Acid in Brain Aging

In the central nervous system, AA plays a complex role that is still only partially established. Cerebrospinal fluid (CSF) ascorbic acid concentrations (200–400 mM) are higher compared to those in cerebral parenchyma and in plasma (30–60 nM) [43].

AA is secreted into the CSF across the apical membrane of choroid plexus cells by an active and saturable transporter for AA, the sodium-dependent vitamin C transporter 2 (SVCT2). In turn, dehydroascorbate (DHA) can cross the blood-brain barrier (BBB) more efficiently through the GLUT1 transporter present in the BBB endothelial cells. SVCT2 mediates AA uptake through neurons or astrocytes in the brain while GLUT receptors (In particular GLUT1 and GLUT3) are primarily responsible for the DHA absorption from the central nervous system cells. Neurons likely use both mechanisms to maintain intracellular ascorbate, although SVCT2 transport mostly contributes to maintaining the ascorbate concentration gradient from CSF to neurons [43]. Moreover, AA recycling acts through a bystander effect by GLUT receptors mediated cellular uptake in pro-oxidative conditions. It favors the intracellular conversion from DHA to AA with increased intracellular accumulation. This bystander effect is responsible for AA recycling activity between neurons and astrocytes and plays a role in the fine balance between pro-oxidative and anti-oxidative status [53].

Recently, it has been shown that AA release mediated by neurons is linked to glutamate metabolism and kinetics in the brain. In particular, Wilson et al. [53] demonstrated that AA extracellular release is the direct consequence of astrocyte swelling mediated by glutamate receptors’ increased sodium uptake. Heightened AA release in the brain and CSF is considered responsible for its antioxidant and neuroprotective mechanism against glutamate excitotoxicity [53].

Several in vivo studies documented that AA plays an antioxidative role, especially after an ischemic event or cerebral reperfusion. AA, at millimolar concentrations, was able to scavenge the superoxide anion, recycling the α-tocopherol within the lipid layers of the cellular membrane [54]. This, in turn, impeded the lipoperoxidation process. In addition, in the CNS, AA participated in several hydroxylation reactions that include the redox activity of Fe^3+^ and Cu^2+^ at dioxigenase sites. In vitro studies on cultured stem cells showed that AA is also implicated in neuronal developmental maturation and in neurotransmission. Lee et al. [55] further demonstrated that AA (at a 200 millimolar concentration) was effective in differentiating neuronal and astrocytes precursors, promoting synaptic maturation.

Using SVTC knockout mice as an in vivo model showed that AA, at lower doses, was able to mediate dendrite formation, increasing post-synaptic electrical potential [56].

AA is essential for the biosynthesis of catecholamines, peptide amination, myelin formation, synaptic function enhancement, along with the neuroprotective activity against glutamate toxicity [53,57]. In particular, AA plays an essential role in neurotransmission, because it is a co-factor of the dopamine beta–hydroxylase enzyme that catalyzes the conversion from dopamine to noradrenaline. AA is considered to modulate cerebral plasticity by orchestrating neurotransmitter balancing in the brain. The main AA-mediated mechanisms impacting neurotransmission could relate to redox modulating activity of the NMDA receptor, supporting a role for AA in counteracting glutamate excitotoxicity [57,58].

It is expected that better understanding of the physiological and molecular mechanisms associated with AA brain recycling and the differential expression of SVCT2 and GLUT receptors could contribute to disentangling the pathogenesis of complex neurodegenerative diseases, such as Alzheimer’s disease and Huntington’s disease.

## 6. Ascorbic Acid and Its Relevance to Alzheimer’s Disease

Over the years, l-ascorbic acid (AA) has been increasingly found to promote several beneficial effects on neurodegeneration, with particular regard to Alzheimer’s disease (AD) [59]. The increasing burden of this life-threatening condition [60] and the lack of disease-modifying drugs have guided the research towards preventive strategies, targeting AD modifiable risk factors [12]. Mounting evidence indicates a role for l-ascorbic acid in ameliorating specific factors linked to AD pathogenesis [61]. Namely, the main mechanisms associated with AA neuroprotection involve the scavenging activity against ROS, the modulation of neuroinflammation, the suppression of the fibrillation of amyloid-beta peptide (Aβ), and the chelation of iron, copper and zinc [62]. The amyloid cascade hypothesis is considered the primary event of AD pathogenesis [63]. The sequential cleavage by gamma and β secretase (BACE1) of the β-amyloid precursor protein (APP) results in the production of the β-amyloid species with neurotoxic oligomer accumulation. Brain accumulation of Aβ1-42 oligomers results in increased neuronal vulnerability to oxidative stress [64,65] neuroinflammation with impairment of synaptic plasticity [66], and neuronal death. Extracellular amyloid plaques are also responsible for the hyperphosphorylation of the cytoskeletal Tau protein [67]. In addition, Aβ oligomers interfere with mitochondrial dynamics [68,69].

Copper, zinc, and iron are present in Aβ plaques due to the presence of metal binding sites [70]. Metals can affect the morphology of Aβ, accelerating fibrillation and cytotoxicity of Aβ [71]. Therefore, redox active copper and iron linked to Aβ can generate hydroxyl radicals via the Fenton reaction, increasing protein and DNA oxidation and lipid peroxidation (MDA) in the AD brain. Metal redox activity also induces the production of AGEs, carbonyls, peroxynitrites, and increased levels of heme oxygenase-1 (HO-1), with decreased cytochrome c oxidase activity [61]. AGEs, through their interaction with receptors for advanced glycation end products (RAGEs), further activate pro-inflammatory pathways with the induction of pro-inflammatory cytokines, such as IL-6 [72]. In addition, lower concentrations of the fluorescent AGE pentosidine were observed in the CSF of AD patients, compared to healthy subjects, in support of a role for altered AGE metabolism in AD pathogenesis [73].

Oxidative stress is generally associated with chronological aging, while aging is the major epigenetic risk factor for AD. Recent evidence has found that oxidative stress plays an essential role in the pre-phase of AD, including mild cognitive impairment [74]. The brain is vulnerable to ROS damage due to neurons’ post-mitotic state with higher oxygen consumption. With respect to lipid peroxidation products, oxidized proteins, and DNA damage, peroxynitrites have been increasingly detected in the AD temporal cortex, as well as oxidation of mitochondrial DNA and nuclear DNA in the parietal cortex [75]. In AD hippocampal neurons and astrocytes, a redox imbalance has been observed with an overexpression of heme oxygenase-1 and increased levels of Cu/Zn superoxide dismutase [76]. The conjugated aromatic ring of tyrosine residues is also a target for free-radical attack, and accumulation of dityrosine and 3-nitrotyrosine has also been reported in the AD brain [77]. Therefore, oxidative stress can directly activate glia with the priming of astrocytes and microglia at the injury site. In turn, the direct contact of activated glial cells with neurons may generate immune mediators (nitric oxide, ROS, pro-inflammatory cytokines, and chemokines) that are neurotoxins, spreading inflammation in the central nervous system [78,79]. Thus, extensive oxidative damage may act as a driver of brain aging, and early accumulation of oxidatively modified biomolecules may constitute the initial steps of AD neurodegeneration.

All of these findings could provide a mechanistic role for oxidative stress as a direct effect of aging and a consequence of the toxic effect of Aβ. Oxidative stress interacts with multiple features associated with AD pathogenesis, such as APP processing, mitochondrial dysfunction, and metal accumulation [80]. The main AA mediated neuroprotective effects on AD pathogenesis are reported and illustrated in Table 2.

### 6.1. Ascorbic Acid and Oxidative Stress in Alzheimer’s Disease

AA is suggested to play a major role in the pathogenesis of AD by direct neuroprotection against oxidative stress. Imbalance of AA homeostasis has been extensively demonstrated in neurodegeneration [58]. AA is a key antioxidant of the CNS, released from glial cells to the synaptic cleft, and taken up by neurons as an antioxidant defense to maintain neuronal metabolism and synaptic function. The astrocyte-neuron interaction was found to function as an essential mechanism for AA recycling, participating in the anti- oxidative defense of the brain [97].

It is well documented that AA is a first-line antioxidant defense to neutralize ROS reactivity, promoting the regeneration of endogenous antioxidants (GSH, catalase, vitamin E) [98]. 

Interestingly, it is also presumed that AA moderates the oxidative stress mediated by glutamate, protecting from excitotoxicity in the brain [56,88]. A previous study in APP/PSEN 1 transgenic mice showed that parenteral administration of AA possessed nootropic properties, without altering the AD-like features of plaque deposition, oxidative stress and acetylcholinesterase activity [85]. Therefore, several in vitro and in vivo studies underpin the therapeutic role of AA in AD, bolstering oxidative defense [99].

In rat hippocampal brains, oral administration of AA reduced oxidative stress and neuroinflammation mediated by Aβ fibrils [100]. Additionally, AA was shown to protect SH-SY5Y neuroblastoma cells from apoptosis mediated by Aβ [86], decreasing the rate of endogenous amyloid generation. Further, AA was reported to decrease acetylcholinesterase activity in mice [101] and to positively restore presynaptic acetylcholine release [102].

More recently, the NO-catalyzed release of anhydromannose in the presence of AA was detected [103] with an associated decreased formation of toxic Aβ oligomers. APP/PSEN 1 mice lacking the SVCT2 transporter and having AA mild deficiency showed accelerated amyloid pathogenesis, linked to oxidative stress pathways, compared to control mice with normal brain ascorbic acid [81]. Further, orally-administered AA reduced oxidative stress and pro-inflammatory cytokines induced by Aβ peptide injections in the CA1 area of the hippocampus in rat brains [99].

### 6.2. Metals, Oxidative Stress and Ascorbic Acid in Alzheimer’s Disease: The AA Oxidative Balance in the Brain

The main features of enhanced oxidative stress in the AD brain are also related to the increased content of Cu and Fe, capable of stimulating free radical generation, lipid peroxidation, reactive nitrogen species (NRS) release, and stress-sensitive proteins [104]. In turn, the interaction of the redox-active copper ions with misfolded Aβ aggregates and oligomers may favor AD pathogenesis.

It is well known that at higher concentrations, AA acts as a pro-oxidant, either by generating reactive oxygen species or by inhibiting the antioxidant systems in the presence of iron, which, in turn, induces lipid peroxidation [105]. A pro-oxidant or antioxidant effect of AA mainly relies on the concentration gradient and redox state of a cell [106]. Evidence from a mouse model that selectively over-expressed the AA transporter SVCT2 in the eye [107] implicated AA in age-related damage to crystalline proteins in the lens. All of these experimental data contribute to heightening the debate on the potential pro-oxidant role of AA via the Fenton reaction. 

A previous study undermined the protective role of AA in dementia, indicating that the interaction of AA with ‘free’ catalytically-active metal ions could contribute to oxidative damage through the production of hydroxyl and alkoxyl radicals [108]. Interestingly, some in vitro studies investigated the pro-oxidant properties of ascorbate [109], which were mainly attributed to the release of metal ions from damaged cells. It has been reported that neurotoxic forms of amyloid β, Aβ (1–42), Aβ (1–40), and also Aβ (25–35) induced copper-mediated oxidation of ascorbate, whereas non-toxic Aβ (40–1) did not [110,111]. It was concluded that toxic Aβ peptides mediated copper-oxidation of ascorbate with the generation of hydroxyl radicals, indicating a role for cupric-amyloid peptide’s free radical generation in the pathogenesis of AD. In line with these last findings, Aβ was not found to silence the redox activity of Cu^2+^/^+^ via chelation, but rather hydroxyl radicals were produced as a result of Fenton-Haber Weiss reactions of ascorbate and Cu^2+^, rapidly quenching harmful radicals [112]. Moreover, reaction rates and mechanisms of AA oxidation resulted in greater biological relevance in the presence of Cu(II)-containing Aβ oligomers and fibrils, given the close proximity of ROS to cell membranes [104]. Further evidence indicated a pro-oxidative role for AA in the interaction of the redox-active copper ions with misfolded amyloid β and AD pathogenesis, with particular relevance to catalytic sites for Cu^+^ present in full-length Aβ instead of in any particular Aβ conformation [82].

However, to complicate the issue, AA was observed to reduce in vivo oxidative damage in the presence of iron, despite its well-known in vitro pro-oxidant properties in buffer systems containing iron [83]. In addition, a recent report evaluated the in vitro effects of different food constituents on brain metal chelation, oxidative stress, and fibrillogenesis [89]. The results did not support the currently hypothesized AA neuroprotective mechanisms of action. Indeed, AA was found to be a good antioxidant with poor metal chelating activity. Strikingly, the study did not show any AA-mediated inhibiting effect on Aβ fibrillogenesis, compared to the multifunctional food abilities of epigallocatechin gallate (EGCG), gallic acid, and curcumin. Hence, due to good AA brain uptake, further investigation is needed to address the role of ascorbic acid in counteracting oxidative stress in an AD brain.

### 6.3. Ascorbic Acid and Neuroinflammation in Alzheimer’s Disease

A previous study demonstrated that chronic administration of AA in the brain chronically infused with lipopolysaccharide and tiorphan was associated with increased deposition of Aβ amyloid plaques and increased Aβ neuronal immunoreactivity [92]. However, a body of evidence implicated AA in the suppression of glia-mediated inflammation. In particular, a colchicine-induced oxidative stress/neuroinflammation AD rat model [84] showed that administration of AA was effective in preventive memory impairment, and reducing inflammatory markers (TNF alpha, IL 1 beta), ROS, and nitrite levels in the hippocampus of AD rats. AA also significantly reduced amyloid plaque formation. Peripheral immune response (increased phagocytic activity of blood WBC and splenic PMN) was also recovered after AA administration and the observed changes were associated with the higher efflux of inflammatory mediators from the brain to peripheral circulation. The same results also addressed a pro-oxidant role of AA at higher doses (600 mg diet), supporting the dual role of AA in addressing the oxidative stress. In addition, a rat model of ethanol-induced oxidative stress showed that AA was effective in counteracting ethanol-induced oxidative stress, neuroinflammation, and apoptotic neuronal loss with beneficial effects against ethanol damage to brain development [87]. Even if the model is not a true AD model, the current findings add knowledge to the role of AA against oxidative stress and neuroinflammation in the brain. In particular, due to its free radical scavenging properties, AA treatment reduced the production of ROS and suppressed both activated microglia and astrocytes. AA also demonstrated mitigation of apoptosis and neurotoxicity by decreasing levels of the Bax/Bcl-2 ratio, cytochrome C, and different caspases, such as caspase-9 and caspase-3. Moreover, AA treatment reduced ethanol-induced activation of poly [ADP-ribose] polymerase 1 (PARP-1) and neurodegeneration. In line with these data, AA was also observed to suppress the lipopolysaccharide (LPS)-stimulated production of inflammatory mediators in neuron/glia co-cultures by inhibiting the MAPK and NF-κB signaling pathways [113].

### 6.4. Ascorbic Acid and Amyloid Plaque Accumulation in Alzheimer’s Disease

Accumulating evidence indicates a role for AA on the toxic fibrillogenesis of Aβ. High doses of AA supplementation reduced the amyloid plaque burden in a 5 familial Alzheimer’s disease mutation (5XFAD) AD mouse model. To better identify the pathogenetic importance of AA in an AD mouse model, the cross-breeding of 5XFAD mice with gulono-gamma-lactone oxidase (Gulo) knockout mice was performed (KO-Tg mice). The higher supplementation of AA in KO-Tg mice resulted in the amelioration of BBB disruption and mitochondrial alteration, with substantial reduction of amyloid plaque burden [114]. 

The APPSWE/PSEN1deltaE9 mouse model of AD, created by crossing APP/PSEN1(+) bigenic mice with SVCT2(+/−) heterozygous knockout mice, also showed interesting results [81]. By 14 months of age, increased oxidative stress was observed (malondialdehyde, protein carbonyls, F2-isoprostanes) with decreased total glutathione, compared to wild-type controls. In addition, increased amounts of both soluble and insoluble Aβ1-42, and a higher Aβ1-42/1-40 ratio with increased hippocampal and cortical amyloid-β plaque deposits were observed, compared to APP/PSEN1(+) mice with normal AA brains. These data suggested that AA deficiency plays an important role in accelerating amyloid accumulation, particularly during early stages of disease, and that these effects are likely modulated by oxidative stress pathways. Huang et al. showed that pre-loading cells with ascorbate substantially prevented apoptosis and death of SH-SY5Y cells, while also decreasing basal rates of endogenous beta-amyloid generation [86]. Cheng et al. demonstrated, in an in vitro model, that an inadequate supply of AA could contribute to the increased formation of toxic Aβ oligomers. In the absence of AA, the temporary interaction between the Aβ domain and small NO-catalyzed release of anhydromannose (anMan)-containing oligosaccharides is prevented, with the increased induction of neurotoxic fibrillogenesis [103]. Murakami et al. [91], in APP transgenic mice, showed that AA administration attenuated oligomerization, but not the total amyloid plaque volume. The authors concluded that the ability of mice to retain de novo synthesis of AA is possible, and a longer study duration is needed to appreciate the significant changes in amyloid plaque accumulation. These last findings are original and indicate the need to test the “sink hypothesis” through the systematic assessment of cerebrospinal fluid AA levels in order to support the role of AA in promoting healthy brain aging. Indeed, it is hypothesized that there is some form of equilibrium for the Aβ in the brain and the periphery such that Aβ can be transported across the blood-brain barrier. By modulating the peripheral Aβ levels, it is predicted that the brain Aβ levels will undergo concomitant changes, forming the basis of the “sink hypothesis” for Aβ lowering strategies.

### 6.5. Acid Ascorbic and Vascular Disease Associated with Alzheimer’s Disease

Recently, a pathophysiological role of the vascular component in the pathogenesis of AD has been demonstrated [115]. Again, oxidative stress is considered a key relevant mediator, confirming the pathogenetic link between AD and vascular disease [116]. Oxidative stress may affect the neurovascular unit, by impairing the endothelial integrity with increased Aβ42 production. This series of pathological events resulted in automatically maintaining the cycle between ROS overproduction and new extracellular Aβ42 deposition. It was ascertained that AA mediates a series of protective effects on brain neurodegeneration by reducing intima-media thickness, lipid peroxidation, and endothelial dysfunction [90,116,117,118,119]. In keeping with this, it has been recently documented that the integrity of the endothelial lining in the blood-brain barrier is essential to prevent the onset of AD [120,121,122]. Each of these vascular risk factors may represent a biological target for AA and contribute to the preventative role of AA in the development of AD pathogenesis, associated with the vascular component.

Growing evidence indicates a role for AA in reducing cardiovascular related mortality and overall mortality, according to higher quartile plasma AA concentrations in humans [123]. Interestingly, it should be noted that the higher risk of carotid intima thickness >1.2 mm was exclusively associated with the lowest plasma AA tertile. This same increased risk was not observed with uric acid, vitamin A, or enzymatic antioxidant load (superoxide dismutase and glutathione oxidase activity). Similarly, dietetic interventions in elderly subjects showed that carotid intima thickness progression was reduced only in those subjects taking AA daily.

The risk of either AD or vascular dementia is higher in patients with elevated blood pressure, which suggests how arterial stiffness and atherosclerosis play important pathogenetic roles [123]. Endothelial dysfunction is associated with arterial stiffness which, in turn, is a strong predictor for cognitive decline [124]. All of these data support the role for AA in modifying vascular risk factors associated with Alzheimer’s-type dementia.

Endothelial dysfunction is a crucial factor associated with AD pathogenesis. Aβ aggregates are cleared from the brain across the BBB, as the transport is finely regulated by RAGE receptors and LDL receptor-related protein (LRP-1). In patients with AD, brain endothelial LRP-1 expression throughout the BBB is reduced [125]. These data suggested an essential role of the endothelial cell integrity lining in the onset and progression of AD. The efficacy of AA against BBB breakdown due to cortical compression was reported [90]. A model of ischemic-reperfusion [126] and BBB breakdown with reduced NO bioavailability may be considered a prototypical model to understand the pleiotropic roles of AA on endothelial function. AA regulates endothelial integrity via oxidative pathways; superoxide generated by endothelial cells reacts with NO to form cytotoxic peroxynitrites and AA could decrease NO consumption by scavenging superoxide. In addition, AA was found to play a role in the function of endothelial nitric oxide synthase (eNOS) by recycling the eNOS co-factor, tetrahydrobiopterin, which is relevant for arterial elasticity and blood pressure regulation [18]. AA also favored the restoration of NO metabolism from *S*-nitrosothiols in plasma [127], reducing nitrite (NO2) to NO, which may preserve NO in tissues or plasma. AA was reported to reverse the generation and metabolism of NO [94], and to prevent endothelial dysfunction by inhibiting LDL oxidation. An oxidized endothelium is known to increase BBB permeability [117] and the AA-associated protective mechanism on lipid metabolism was found to improve BBB endothelial disruption. In addition, AA prevented the impaired response to the vasodilator acetylcholine (endothelium-dependent agonist) and reduced ROS (e.g., superoxide) produced by neutrophils [93]. Thus, a series of studies have suggested that AA may protect from AD onset, by protecting BBB integrity.

So far, scant investigations have explored the effects of anti-oxidative vitamins on dementia through the cerebrovascular axis [95]. The study of Kook et al. [87] recently reported that high-dose supplementation of AA reduced amyloidosis in AD mice (5XFAD) via the reduction of BBB disruption and mitochondrial alteration [96]. Additionally, AA was also reported to prevent the disruption of BBB by upregulating the expression of tight junction proteins, occludin and claudin-5.

In a model of stroke with substantial BBB disruption, AA significantly reduced BBB permeability [128]. Similarly, in a mouse model of cerebral ischemia, AA ameliorated BBB dysfunction by reversing tight junction claudin-5 and attenuated edema and neuronal loss [129]. Moreover, an in vitro study provided evidence that AA reversed hyperglycemia-mediated BBB disruption [130]. To date, AA seems to offer neuroprotection by restoring BBB integrity. However, further investigations should focus on simultaneously testing brain neuroprotective effects and BBB protective effects of antioxidants [95]. AA seems to possess both types of neuroprotection and could be further tested as a targeted dual agent for preventing cognitive decline.

Several cross-sectional studies have demonstrated a lower CSF-to-plasma AA ratio in AD patients compared to controls. In particular, recent findings [95] suggest that maintenance of a high CSF-to-plasma AA ratio is important in preventing cognitive decline in AD and that BBB impairment unfavorably affects this ratio. However, whether the AA transport carrier dysfunction (SVCT2) or the disturbed BBB integrity is responsible for it, is still a matter of debate.

Indeed, the loss of BBB integrity seen in elderly people with dementia may hamper the brain’s ability to retain CNS AA regardless of the successful transport [131,132,133]. Genetic variations of the SVCT2 carrier at the choroid plexus and in neurons may also play a significant role. In line with these data, a recent review has concluded that CSF levels within the normal range for AA indicate the preservation of choroid plexus function and AA transport into the CSF [134], despite lower plasma levels.

## 7. From Bench to Bedside

Ascorbic acid levels in plasma are decreased in AD patients [135] and the association between cognitive impairment and low antioxidant status is accumulating. Indeed, it has been suggested that increased dietary intake may reduce the risk of developing AD. So far, whether oxidative stress associated with the disease is responsible for the reduction of antioxidants, or whether the low antioxidants contribute to the progression of the disease has not been ascertained.

However, plasma levels of AA were found to be lower both in patients with mild cognitive impairment and AD, compared to controls [136].

A further difference existed between undernourished patients with dementia and patients taking any antioxidant supplement. 

From bench to bedside, eight large population studies [57] have investigated the association between AA intake and Alzheimer’s-type dementia in both European countries and the US. However, the neuroprotection associated with AA has not yet been established. According to the CHAP study [137], none of the elderly dementia-free participants longitudinally developed dementia, due to AA supplementation. In contrast, a synergistic association between AA and vitamin E supplementation was shown in reducing the risk of AD [138]. Another large population study has shown a protective role for AA in vascular dementia and cognitively-intact subjects, but no protective role of AA was shown for Alzheimer’s disease [139].

The Rotterdam study showed the most consistent association between higher AA intake and reduced relative risk for AD in the largest population study [140] with the higher magnitude of association in people most depleted of AA (e.g., smokers). The same study found an association for lower levels of vitamin E in AD patients at follow up an average of 9.6 years later, but not for AA intake.

Additionally, eleven studies have examined the relationship between plasma AA and cognitive decline, including AD, and four of them examined CSF AA and CSF-to-plasma AA ratios. An early study of Goodwin [29] assigned patients to AA plasmatic deficiency according to tertiles. The main findings suggested a significant association between AA deficiency below 20 µM, mild cognitive impairment, and AD patients compared to healthy controls, even after correction for co- morbidities, age, and fruit/vegetables intake.

The study of Quinn [141] showed that the mean CSF-to-plasma AA ratio was significantly lower in AD compared with controls. A further prospective analysis of CSF AA, rates of cognitive decline, and BBB did not draw final conclusions, but a higher CSF-to-plasma AA ratio was associated with a slower rate of decline [57].

Conversely, several clinical studies did not show any beneficial effect of AA on cognition in patients with AD. In a population study of North Carolina, 616 elders aged over 75 years and long-term supplement users of AA did not show any neuroprotection against developing AD [142]. However, no record was made of dietary intake, and the results outlined that less healthy behaviors and socioeconomic status were associated with the poorer cognitive outcomes. Vitamin E levels were significantly lower in the AD group than controls while AA levels did not differ significantly between groups.

A series of limitations may count for interpreting the clinical results. There is substantial inconsistency among the observational studies on AA intake, plasma levels, beneficial effects on aging and cognition.

It is noteworthy that a great deal of clinical studies usually excluded elderly people if they had polypharmacy or comorbidity. These exclusion criteria critically undermine the validity of results, leaving out the populations most at risk for AA deficiency.

Approximately 17% of the elderly population did not meet the RDA for AA intake, which critically suggests that large elderly population groups show depleted levels of AA; this determinant may count for the disparate conclusions of different clinical studies. In particular, as accurately summarized by Harrison F et al. [143], the classification of groups according to AA status differs greatly among studies. The deficiency levels of AA are lower than 11 µmol/L, with suboptimal concentrations between 11–38 µmol/L, adequate plasmatic concentrations of AA above 28 µmol/L and optimal concentrations between 50–60 µmol/L (µmol/L: conversion factor 56.78 from mg/dL concentrations of AA).

Moreover, the range of AA supplements greatly vary from 27 to 230–270 mg/day, introducing another element of variability. 

Not least, the higher intakes of AA were associated with beneficial effects on cognition if not exceeding 500 mg/day; higher plasmatic values (1 g/day) of AA were associated with poorer cognitive performance.

The beneficial effects of AA need reliable plasmatic determinations with repeated points of measurement. The lack of accuracy in study designs and methodologies may also affect the reliability of the findings [57]. In addition, the lack of standardization between single nutrient consumption or multivitamins and the lack of systematic detection of plasmatic levels of AA also affect the accuracy of outcomes.

Moreover, the missing consideration of specific AA metabolism, the inaccurate daily estimate of AA consumption, and the erroneous intestine-to-bloodstream absorption due to saturable AA transporters being critical determinants for drawing appropriate conclusions [57]. The variability of the results may also be ascribed to difference of plasma AA concentrations according to polymorphisms of SVCT2 and SVCT1 despite equivalent AA intake. This difference indicates that SVCT1/2 genotype may play an important role in the association between AA intake and circulating AA levels. Furthermore, the difference between food intake and synthetic supplements and the mean of their bioavailability need to be clearly defined.

It should also be noted that the current intake of AA may not accurately reflect the subjects’ lifetime habits; this, in turn, may substantially affect the biological trajectory of Alzheimer’s disease with particular regard to early mid-life deposition of amyloid plaque. Thus, the stratification of different aging populations according to their clinical vulnerability, cognitive reserve, comorbidity and specific risk profile could add knowledge to this field [95]. Similarly, a greater understanding of the modulation between pro-oxidative AA status and antioxidative capacity during aging and age-related specific conditions, including dementia, is warranted. 

Currently, the clinical data yield inconsistent results. AA supplementation showed beneficial effects when restoring a nutritional deficit or preventing vitamin deficiency; thus, it seems more plausible that avoiding AA deficiency is likely to be more beneficial than taking supplements on top of a normal healthy diet.

So far, the levels of AA needed to beneficially modify brain aging are largely unknown. A causal association between AA deficiency and cognitive decline, including dementia, is still debated and two main issues are unanswered. Namely, the co-causal role of AA deficiency versus its epiphenomenal role in AD neurodegeneration has not yet been established. However, AA’s strong free radical scavenging properties, the well-characterized kinetics of transport, and the good bioavailability in the CNS provide a favorable background for further exploring its role in promoting brain function and healthy aging.

With testing by neuroimaging, recent research has demonstrated that it is possible to detect brain levels of AA by using a MEGA-PRESS mediated spectra (MEGA-PRESS, MEGA-point-resolved spectroscopy) [144]. The study indicated a relationship between brain and blood AA levels and provides a new conceptual framework for future studies, further exploring the role of AA in the brain.

## 8. Conclusions

In conclusion, randomized clinical trials have failed to demonstrate any association between AA-mediated antioxidant therapeutic activity and a delay in AD neurodegeneration.

However, the assessment of the “sink hypothesis’’ could substantiate a crucial role for AA in promoting healthy aging of the brain. The analysis of AA concentration in plasma, CSF, and the ratio of AA/glutamate, along with the role of AA-related carriers (SVCT2 SNPs) and barriers to its brain transport (BBB) could significantly spur this research field by directly analyzing the AA concentrations in the brain.

Neuroimaging measures also hold promise in offering deeper insights on the structural, metabolic, and connective role that AA plays in the brain [144], contributing to the larger picture.

Additionally, animal models need to be further investigated with a particular focus on gulonolactone oxidase knockout models that mimic human physiology, and may help identify novel AA mechanisms of action to promote healthy aging of the brain.

Another intriguing area of research could address the protective association between AA and glutamate transport/NMDA receptors to critically evaluate the AA role in neurodegeneration.

Finally, the field of epigenetics has recently answered the question as to why AA is disproportionally concentrated in CSF and brain parenchyma compared to plasma [28]. Nutrition represents one of the most powerful environmental modifications of the genome. Recent research has assessed a peculiar epigenetic role for AA. Namely, the oxidation of 5-mc (5-methylcytosine) to 5-hmc (5-hydroxymethylcytosine), as part of dynamic DNA demethylation, is catalyzed by TET (ten-eleven translocation) dioxygenase enzymes, for which AA is a critical co-factor [23,145]. Interestingly, no other antioxidant displayed such an epigenetic mechanism. Thus, AA can be considered vital for neuronal repair and offers new molecular mechanisms to understand the true neuroprotective role of AA in brain aging and neurodegeneration.

In addition, it has been recently documented that 75% of ascorbic acid degradation is due to Maillard degradation pathways (amide-AGEs) [146]. Knowledge of the mechanisms of Maillard model systems could help understand the changes occurring during storage and processing of AA-containing food, as well as in vivo modifications.

Thus, all of these lines of research could improve the understanding of the role of AA in brain aging and, hopefully, provide a new conceptual framework for AD in the near future.

## Figures and Tables

**Table 1:** In vitro and in vivo evidence for a role of AA in the aging process.

Species	Model	Design and Methods	Conclusion	References
Human	ESCs	Methylation	Epigenetic regulation of Tet activity and DNA methylation	Blaschke, K. 2013 [28]
Mouse	Embryonic fibroblasts cultured	Expression of Tet genes, GSH antioxidant activity	Epigenetic modulation of genome activity and stability	Minor, E.A. [29]
Human	Umbilical cord vein endothelial cells (HUVEC)	Measurement of citrulline synthesis, determination of cGMP, eNOS activity, GTP Cyclohydrolase I	Anti-oxidative pathways (protection of tetrahydrobiopterin Endothelial integrity (cellular NO synthesis))	Heller, R. 2001 [18]
In Vitro	EA. hy926	Determination of BH4 levels, H_2_O_2_, expression of PP2Ac	Endothelial integrity (eNOS activity/eNOS phosphorylation)	Ladurner, A. 2012 [19]
Mouse	WrnΔhel/Δhel	Measurement ROS and oxidative DNA damage	Extended life span, improvement of inflammation, metabolic profile, lipid profile	Lebel, M. 2010 [30]
Mouse	Gulo−/−	Measurement cytokines and metabolites	Extended life span. Model of rejuvenation	Aumailley, L. 2016 [31]
Insect	Wrn-1(gk99) mutant	Gene expression and regulation	Extended mean life span of *C. elegans* (regulatory genes of lipid metabolism, ketones, organic acids, carboxylic acids. Locomotion and developmental anatomical structure)	Dallaire, A. 2014 [32]
In Vitro	WS MSC model	Oxidative stress levels, IL-6 anIL-8	Model of rejuvenation	Li, Y. 2016 [33]
Mouse	WrnΔhel/Δhel	Measurements of ROS and oxidative DNA damage, GSH, ATP, protein analysis, lactonase activity	Beneficial effects on oxidative pathways, genome stability	Massip, L. 2010 [34]
Human and Mouse	PBMCs and BMMC and BM from SOD1−/−	PBMC: IFN-γ ± NAC Human and mouse BM: BFA ± NAC	Beneficial effects on immunosenescence through inflamm-aging	Pangrazzi, L. 2016 [35]
Mouse and In Vitro	3T3-L1 cells, OP9 cells	Adipocytes differentiation	Adipocyte differentiation: implications for the aging process	Rahaman, F. 2014 [36]
Mouse	Embryonic stem cell line CGR8	Stem cells differentiation	AA-dependent differentiation (p38 MAPK/CREB pathway). Epigenetic regulation	Rahman, F. 2016 [37]
Human In Vitro	hBM-MSCs	Osteocyte and adipocyte differentiation	Beneficial effects on cells differentiation mediated by anti-oxidation	Jeong, S.G. 2015 [38]
Mouse	WrnΔhel/Δhel	Metabolite, cytokine and chemokine measurements	Potential predictive cardiometabolic biomarkers in patients with WS.	Aumailley, L. 2015 [39]
Mouse	SMP30KO	Immunosenescence and aging	Beneficial effect on the maintenance of immune cells (thymic atrophy)	Uchio, R. 2015 [40]
Mouse	SMP30/GNL KO	Model of senescence	Beneficial effects on liver protein oxidation in vivo	Sato, Y. 2014 [41]
In Vitro	Cortical precursor cells	Survival, proliferation, and differentiation of AA-treated CNS precursor cells	Brain development: the generation of neurons and glia	Lee, J.Y. 2003 [42]
Mouse	Hippocampal and cortical neurons from mice lacking one allele of the SVCT2	Combined treatment of AA and GSH	Beneficial effects on neuronal development, functional maturation, and antioxidant responses	Qiu, S. 2007 [43]

**Table 2:** In vitro and in vivo evidence for a role of AA in Alzheimer’s disease.

Species	Model	Design and Methods	Conclusion	References
Mouse	TASTPM	Evaluation of carbonyls, glutathione, Αβ, APP	Decreased oxidative stress markers, Nrf2, GSH, APP, soluble Aβ1-42. No increase of BACE 1, PS1and AB plaque	Choundhry, F. 2012 [58]
Mouse	Model with human APP695 and double mutation (K670N, M671L)	Evaluation of Aβ, BACE1, antioxidant system and IL-1β	Increased antioxidant system, reduced activity of BACE, IL-1β and NO levels, Aβ deposition	Apelt, J. 2004 [60]
Mouse	APP/PS1 transgenic	ROS scavengers and inhibitors effects on Aβ-induced impairments in LTP	Reversal of Aβ- deposition by mitochondria-targeted ROS scavenging	Ma, T. 2011 [62]
Mouse	HAPP/Sod1−/−	Anti-Aβ1-16 antibody	Inhibition of amyloid plaques (Aβ hexamers /BACE1 modulation)	Murakami, K. 2012 [65]
Mouse	Tg2576	Aβ levels brain deposition	Suppressed brain inflammatory and oxidative stress responses in mice, significant reduction of soluble and insoluble Aβ1-40 and Aβ1-42	Yao, Y. 2004 [57]
Mouse	APPS we/PSEN1ΔE9	MDA, Aβ levels, AChE activity. Learning and memory	Improvement of learning and memory Beneficial effects against MDA, and Beneficial effects on AChE function	Harrison, F.E. 2009 [50]
Mouse	APPSWE/PSEN1ΔE9 mice, SVCT2+/−	Behavioural test, GSH, MDA, isoprostanes	Decreased Aβ deposition (senile plaque formation and accumulation)	Dixit, S. 2015 [81]
Rat	F-344	Aβ deposition	Decreased-amyloid immunoreactive fibrils	Hauss-Wegrzyniak, B. 2002 [82]
Rat and Mouse	Charles-Foster, Swiss Albino mouse	Cognitive test, cytokines, ROS Cytotoxic Activity Assay	Enhancement of anti-oxidative pathway	Sil, S. 2016 [83]
Mouse	Gulo−/−5XFAD	Identification modification of cerebral capillaries	Reduction of Aβ accumulation	Kook, S.Y. 2014 [84]
In Vitro	neuroblastoma cell line SH-SY5Y	Apoptosis (phosphatidylserine, TUNEL assay, caspase-3 activity)	Prevention of toxicity induced by Aβ	Huang, J. 2006 [85]
Mouse	Tg2576, 3xTg-AD	Aβ staining, investigation APP and HS oligosaccharides	Modulation of Aβ fibrillogenesis	Cheng, F. 2011 [86]
Mouse	AD model	Fibrillogenesis: senile plaques	Modulation of synaptophysin and the phosphorylation of tau at Ser396	Murakami, K. 2011 [87]
Rat	Wistar	Lipoperoxidation, oxidation, Inflammation, nitrites	Reduction of pro-inflammatory cytokine Inhibition of Aβ deposition	Rosales-Corral, S. 2003 [88]
Human In Vitro	NT2 undifferentiated cells	Measurement levels of Aβx-40 and Aβx-42, HNE, expression of BACE-1. Evaluation apoptotic cell death induced by HNE	Increased anti-oxidative pathways against SAPK pathways and BACE-1 that regulate AβPP processing	Tamagno, E. 2005 [74]
Human In Vitro	Neuroblastoma cell line SH-SY5Y	Glutathione, superoxide dismutase, and catalase	Neuroprotection anti-oxidative pathways Improvement of antioxidant defense system	Ballaz, S. 2013 [80]
Rat	PND7	Induction of ROS, apoptotic markers. Quantification of Bax/Bcl-2 ratio, cytochrome c and caspases	Reduction of oxidation, neuroinflammation (both activated microglia and astrocytes). reduced ethanol-induced activation of PARP-1 and neurodegeneration	Ahmad, A. 2016 [89]
In Vitro	EA. hy926 cells	Measurement intracellular ascorbate and GSH	Endothelial integrity (NO: eNOS/guanylate cyclase pathway)	May, J.M. 2011 [90]
In Vitro	EA. hy926 cells	Quantification LDL-enriched lipoproteins, GSH, and lipid peroxidation	Endothelial integrity	May, J.M. 2010 [91]
Rat	Cortical neuron/glia co-cultures of neonatal	Measuring nitrites IL-6 and MIP-2, LDH. p38 and ERK MAPKs	Suppression of the LPS-stimulated production of inflammatory mediators	Huang, Y.N. 2014 [92]
Rat	Sprague–Dawley	Behavioural test BBB components	Modulation of cortical compression and/or BBB dysfunction	Lin, J.L. 2010 [12]
Rat	MCAO	Measurement of infarct and edema brain, measurement of serum MMP-9 levels, behavioural testing	decreased MMP-9 levels, Improvement of the vascular insult (BBB disruption and brain edema)	Allahtavakoli, M. 2015 [93]
Rat	Brains	Assessment the role of nanocapsulated ascorbic acid (NAA)	NAA exerted protection to brain mitochondria by preventing oxidative damage in ROS mediated CIR injury	Sarkar, S. 2016 [94]
Rat	Hippocampal neurons	Incubation with Aβ Os or 4-CMC ± NAC	NAC prevention of Aβ O-induced mitochondrial Fragmentation by anti-oxidative pathways	Sanmartin, C.D. 2012 [61]
Rat	Cortical neurons Neuroblastoma cells	Oxidative stress and DHA uptake, analysis of GLUTs	Improvement of anti-oxidative defense of neurons	García-Krauss, A. 2016 [75]
Rat	Primary neurons	Incubation with H_2_O_2_, ratio GSH/GSSG	Increased glutathione system of peroxide detoxification	Dringen, R. 1999 [78]
Rat	Astroglial cells	Treatment with H_2_O_2_ or hydro peroxide, NO release, Lipid Peroxidation, ROS	Reduction of neuroinflammation (microglial-astroglial cells)	Röhl, C. 2010 [79]
Rat	SD	Induction of transient focal cerebral ischemia, treatment with DHA	DHA reduced brain edema and vascular permeability formation following cerebral ischemia	Song, J. 2015 [95]
Human	Endothelial cell (HBMEC) and astrocyte co-colture	BBB after hyperglycaemic insult	Improvement of BBB permeability by reducing oxidative stress associated with glucose normalization	Allen, C.L. 2009 [96]

## References

[1] Bloom D.E. (2011). 7 billion and counting. Science.

[2] Cannizzo E.S., Clement C.C., Sahu R., Follo C., Santambrogio L. (2011). Oxidative stress, inflamm-aging and immunosenescence. J. Proteom..

[3] Franceschi C., Campisi J. (2014). Chronic inflammation (inflammaging) and its potential contribution to age-associated diseases. J. Gerontol. Ser. A Biol. Sci. Med. Sci..

[4] Vasto S., Candore G., Balistreri C.R., Caruso M., Colonna-Romano G., Grimaldi M.P., Listi F., Nuzzo D., Lio D., Caruso C. (2007). Inflammatory networks in ageing, age-related diseases and longevity. Mech. Ageing Dev..

[5] Cevenini E., Monti D., Franceschi C. (2013). Inflamm-ageing. Curr. Opin. Clin. Nutr. Metab. Care.

[6] Michaud M., Balardy L., Moulis G., Gaudin C., Peyrot C., Vellas B., Cesari M., Nourhashemi F. (2013). Proinflammatory cytokines, aging, and age-related diseases. J. Am. Med. Dir. Assoc..

[7] Franceschi C., Bonafe M., Valensin S., Olivieri F., De Luca M., Ottaviani E., De Benedictis G. (2000). Inflamm-aging. An evolutionary perspective on immunosenescence. Ann. N. Y. Acad. Sci..

[8] Szarc vel Szic K., Declerck K., Vidakovic M., Vanden Berghe W. (2015). From inflammaging to healthy aging by dietary lifestyle choices: Is epigenetics the key to personalized nutrition?. Clin. Epigenetics.

[9] Santoro A., Pini E., Scurti M., Palmas G., Berendsen A., Brzozowska A., Pietruszka B., Szczecinska A., Cano N., Meunier N. (2014). Combating inflammaging through a Mediterranean whole diet approach: The nu-age project’s conceptual framework and design. Mech. Ageing Dev..

[10] Berendsen A., Santoro A., Pini E., Cevenini E., Ostan R., Pietruszka B., Rolf K., Cano N., Caille A., Lyon-Belgy N. (2014). Reprint of: A parallel randomized trial on the effect of a healthful diet on inflammageing and its consequences in European elderly people: Design of the nu-age dietary intervention study. Mech. Ageing Dev..

[11] Neufcourt L., Assmann K.E., Fezeu L.K., Touvier M., Graffouillere L., Shivappa N., Hebert J.R., Wirth M.D., Hercberg S., Galan P. (2015). Prospective association between the dietary inflammatory index and metabolic syndrome: Findings from the su.Vi.Max study. Nutr. Metab. Cardiovasc. Dis. NMCD.

[12] Padayatty S.J., Katz A., Wang Y., Eck P., Kwon O., Lee J.H., Chen S., Corpe C., Dutta A., Dutta S.K. (2003). Vitamin C as an antioxidant: Evaluation of its role in disease prevention. J. Am. Coll. Nutr..

[13] Naidu K.A. (2003). Vitamin C in human health and disease is still a mystery? An overview. Nutr. J..

[14] Michels A.J., Joisher N., Hagen T.M. (2003). Age-related decline of sodium-dependent ascorbic acid transport in isolated rat hepatocytes. Arch. Biochem. Biophys..

[15] Grosso G., Bei R., Mistretta A., Marventano S., Calabrese G., Masuelli L., Giganti M.G., Modesti A., Galvano F., Gazzolo D. (2013). Effects of vitamin c on health: A review of evidence. Front. Biosci..

[16] Duarte T.L., Lunec J. (2005). Review: When is an antioxidant not an antioxidant? A review of novel actions and reactions of vitamin C. Free Radic. Res..

[17] Regine H., Gabriele W.-F., Ernst R.W. (2006). Antioxidants and endothelial nitric oxide synthesis. Eur. J. Clin. Pharmacol..

[18] Heller R., Unbehaun A., Schellenberg B., Mayer B., Werner-Felmayer G., Werner E.R. (2001). l-ascorbic acid potentiates endothelial nitric oxide synthesis via a chemical stabilization of tetrahydrobiopterin. J. Biol. Chem..

[19] Ladurner A., Schmitt C.A., Schachner D., Atanasov A.G., Werner E.R., Dirsch V.M., Heiss E.H. (2012). Ascorbate stimulates endothelial nitric oxide synthase enzyme activity by rapid modulation of its phosphorylation status. Free Radic. Biol. Med..

[20] Halliwell B. (2001). Vitamin C and genomic stability. Mutat. Res..

[21] Camarena V., Wang G. (2016). The epigenetic role of vitamin C in health and disease. Cell. Mol. Life Sci. CMLS.

[22] Young J.I., Zuchner S., Wang G. (2015). Regulation of the epigenome by vitamin C. Annu. Rev. Nutr..

[23] Yin R., Mao S.Q., Zhao B., Chong Z., Yang Y., Zhao C., Zhang D., Huang H., Gao J., Li Z. (2013). Ascorbic acid enhances Tet-mediated 5-methylcytosine oxidation and promotes DNA demethylation in mammals. J. Am. Chem. Soc..

[24] Cahill L.E., El-Sohemy A. (2009). Vitamin c transporter gene polymorphisms, dietary vitamin C and serum ascorbic acid. J. Nutr. Nutr..

[25] Langlois M.R., Martin M.E., Boelaert J.R., Beaumont C., Taes Y.E., De Buyzere M.L., Bernard D.R., Neels H.M., Delanghe J.R. (2000). The haptoglobin 2-2 phenotype affects serum markers of iron status in healthy males. Clin. Chem..

[26] Horska A., Mislanova C., Bonassi S., Ceppi M., Volkovova K., Dusinska M. (2011). Vitamin C levels in blood are influenced by polymorphisms in glutathione *S*-transferases. Eur. J. Nutr..

[27] Schwartz B. (2014). New criteria for supplementation of selected micronutrients in the era of nutrigenetics and nutrigenomics. Int. J. Food Sci. Nutr..

[28] Blaschke K., Ebata K.T., Karimi M.M., Zepeda-Martinez J.A., Goyal P., Mahapatra S., Tam A., Laird D.J., Hirst M., Rao A. (2013). Vitamin C induces Tet-dependent DNA demethylation and a blastocyst-like state in ES cells. Nature.

[29] Goodwin J.S., Goodwin J.M., Garry P.J. (1983). Association between nutritional status and cognitive functioning in a healthy elderly population. JAMA.

[30] Lebel M., Massip L., Garand C., Thorin E. (2010). Ascorbate improves metabolic abnormalities in *Wrn* mutant mice but not the free radical scavenger catechin. Ann. N. Y. Acad. Sci..

[31] Aumailley L., Warren A., Garand C., Dubois M.J., Paquet E.R., Le Couteur D.G., Marette A., Cogger V.C., Lebel M. (2016). Vitamin C modulates the metabolic and cytokine profiles, alleviates hepatic endoplasmic reticulum stress, and increases the life span of gulo−/− mice. Aging.

[32] Dallaire A., Proulx S., Simard M.J., Lebel M. (2014). Expression profile of *Caenorhabditis elegans* mutant for the Werner syndrome gene ortholog reveals the impact of vitamin C on development to increase life span. BMC Genom..

[33] Li Y., Zhang W., Chang L., Han Y., Sun L., Gong X., Tang H., Liu Z., Deng H., Ye Y. (2016). Vitamin C alleviates aging defects in a stem cell model for Werner syndrome. Protein Cell.

[34] Massip L., Garand C., Paquet E.R., Cogger V.C., O’Reilly J.N., Tworek L., Hatherell A., Taylor C.G., Thorin E., Zahradka P. (2010). Vitamin C restores healthy aging in a mouse model for Werner syndrome. FASEB J..

[35] Pangrazzi L., Meryk A., Naismith E., Koziel R., Lair J., Krismer M., Trieb K., Grubeck-Loebenstein B. (2016). “Inflamm-aging” influences immune cell survival factors in human bone marrow. Eur. J. Immunol..

[36] Rahman F., Al Frouh F., Bordignon B., Fraterno M., Landrier J.F., Peiretti F., Fontes M. (2014). Ascorbic acid is a dose-dependent inhibitor of adipocyte differentiation, probably by reducing camp pool. Front. Cell Dev. Biol..

[37] Rahman F., Bordignon B., Culerrier R., Peiretti F., Spicuglia S., Djabali M., Landrier J.F., Fontes M. (2016). Ascorbic acid drives the differentiation of mesoderm-derived embryonic stem cells. Involvement of p38 MAPK/CREB and SVCT2 transporter. Mol. Nutr. Food Res..

[38] Jeong S.G., Cho G.W. (2015). Endogenous ROS levels are increased in replicative senescence in human bone marrow mesenchymal stromal cells. Biochem. Biophys. Res. Commun..

[39] Aumailley L., Dubois M.J., Garand C., Marette A., Lebel M. (2015). Impact of vitamin C on the cardiometabolic and inflammatory profiles of mice lacking a functional Werner syndrome protein helicase. Exp. Gerontol..

[40] Uchio R., Hirose Y., Murosaki S., Yamamoto Y., Ishigami A. (2015). High dietary intake of vitamin c suppresses age-related thymic atrophy and contributes to the maintenance of immune cells in vitamin C-deficient senescence marker protein-30 knockout mice. Br. J. Nutr..

[41] Sato Y., Amano A., Kishimoto Y., Takahashi K., Handa S., Maruyama N., Ishigami A. (2014). Ascorbic acid prevents protein oxidation in livers of senescence marker protein-30/gluconolactonase knockout mice. Geriatr. Gerontol. Int..

[42] Kim S.M., Lim S.M., Yoo J.A., Woo M.J., Cho K.H. (2015). Consumption of high-dose vitamin C (1250 mg per day) enhances functional and structural properties of serum lipoprotein to improve anti-oxidant, anti-atherosclerotic, and anti-aging effects via regulation of anti-inflammatory microrna. Food Funct..

[43] Harrison F.E., May J.M. (2009). Vitamin c function in the brain: Vital role of the ascorbate transporter SVCT2. Free Radic. Biol. Med..

[44] Zheng C., Sui B., Hu C., Jin Y. (2015). Vitamin c promotes in vitro proliferation of bone marrow mesenchymal stem cells derived from aging mice. J. Sourn Med. Univ..

[45] Chen B.Y., Wang X., Chen L.W., Luo Z.J. (2012). Molecular targeting regulation of proliferation and differentiation of the bone marrow-derived mesenchymal stem cells or mesenchymal stromal cells. Curr. Drug Targets.

[46] Yang W., Hekimi S. (2010). A mitochondrial superoxide signal triggers increased longevity in *Caenorhabditis elegans*. PLoS Biol..

[47] Van Raamsdonk J.M., Hekimi S. (2012). Superoxide dismutase is dispensable for normal animal lifespan. Proc. Natl. Acad. Sci. USA.

[48] Desjardins D., Cacho-Valadez B., Liu J.L., Wang Y., Yee C., Bernard K., Khaki A., Breton L., Hekimi S. (2017). Antioxidants reveal an inverted U-shaped dose-response relationship between reactive oxygen species levels and the rate of aging in *Caenorhabditis elegans*. Aging Cell.

[49] Riscuta G. (2016). Nutrigenomics at the interface of aging, lifespan, and cancer prevention. J. Nutr..

[50] Soysal P., Isik A.T., Carvalho A.F., Fernandes B.S., Solmi M., Schofield P., Veronese N., Stubbs B. (2017). Oxidative stress and frailty: A systematic review and synthesis of the best evidence. Maturitas.

[51] Leelarungrayub J., Laskin J.J., Bloomer R.J., Pinkaew D. (2016). Consumption of star fruit juice on pro-inflammatory markers and walking distance in the community dwelling elderly. Arch. Gerontol. Geriatr..

[52] Leelarungrayub J., Yankai A., Pinkaew D., Puntumetakul R., Laskin J.J., Bloomer R.J. (2016). A preliminary study on the effects of star fruit consumption on antioxidant and lipid status in elderly Thai individuals. Clin. Interv. Aging.

[53] Nualart F., Mack L., Garcia A., Cisternas P., Bongarzone E.R., Heitzer M., Jara N., Martinez F., Ferrada L., Espinoza F. (2014). Vitamin C transporters, recycling and the bystander effect in the nervous system: SVCT2 versus gluts. J. Stem Cell Res. Ther..

[54] Rice M.E. (2000). Ascorbate regulation and its neuroprotective role in the brain. Trends Neurosci..

[55] Lee J.Y., Chang M.Y., Park C.H., Kim H.Y., Kim J.H., Son H., Lee Y.S., Lee S.H. (2003). Ascorbate-induced differentiation of embryonic cortical precursors into neurons and astrocytes. J. Neurosci. Res..

[56] Qiu S., Li L., Weeber E.J., May J.M. (2007). Ascorbate transport by primary cultured neurons and its role in neuronal function and protection against excitotoxicity. J. Neurosci. Res..

[57] Harrison F.E., Bowman G.L., Polidori M.C. (2014). Ascorbic acid and the brain: Rationale for the use against cognitive decline. Nutrients.

[58] Covarrubias-Pinto A., Acuna A.I., Beltran F.A., Torres-Diaz L., Castro M.A. (2015). Old things new view: Ascorbic acid protects the brain in neurodegenerative disorders. Int. J. Mol. Sci..

[59] Feng Y., Wang X. (2012). Antioxidant therapies for Alzheimer’s disease. Oxid. Med. Cell. Longev..

[60] Ientile L., De Pasquale R., Monacelli F., Odetti P., Traverso N., Cammarata S., Tabaton M., Dijk B. (2013). Survival rate in patients affected by dementia followed by memory clinics (UVA) in Italy. J. Alzheimers Dis..

[61] Yao Y., Chinnici C., Tang H., Trojanowski J.Q., Lee V.M., Pratico D. (2004). Brain inflammation and oxidative stress in a transgenic mouse model of Alzheimer-like brain amyloidosis. J. Neuroinflamm..

[62] Choudhry F., Howlett D.R., Richardson J.C., Francis P.T., Williams R.J. (2012). Pro-oxidant diet enhances beta/gamma secretase-mediated APP processing in APP/PS1 transgenic mice. Neurobiol. Aging.

[63] McGeer P.L., McGeer E.G. (2013). The amyloid cascade-inflammatory hypothesis of Alzheimer disease: Implications for therapy. Acta Neuropathol..

[64] Apelt J., Bigl M., Wunderlich P., Schliebs R. (2004). Aging-related increase in oxidative stress correlates with developmental pattern of beta-secretase activity and beta-amyloid plaque formation in transgenic Tg2576 mice with Alzheimer-like pathology. Int. J. Dev. Neurosci. Off. J. Int. Soc. Dev. Neurosci..

[65] Sanmartin C.D., Adasme T., Hidalgo C., Paula-Lima A.C. (2012). The antioxidant *N*-acetylcysteine prevents the mitochondrial fragmentation induced by soluble amyloid-beta peptide oligomers. Neuro-Degener. Dis..

[66] Ma T., Hoeffer C.A., Wong H., Massaad C.A., Zhou P., Iadecola C., Murphy M.P., Pautler R.G., Klann E. (2011). Amyloid beta-induced impairments in hippocampal synaptic plasticity are rescued by decreasing mitochondrial superoxide. J. Neurosci. Off. J. Soc. Neurosci..

[67] Bloom G.S. (2014). Amyloid-beta and tau: The trigger and bullet in Alzheimer disease pathogenesis. JAMA Neurol..

[68] Moreira P.I., Santos M.S., Oliveira C.R. (2007). Alzheimer’s disease: A lesson from mitochondrial dysfunction. Antioxid. Redox Signal..

[69] Murakami K., Shimizu T. (2012). Cytoplasmic superoxide radical: A possible contributing factor to intracellular abeta oligomerization in Alzheimer disease. Commun. Integr. Biol..

[70] Bush A.I., Masters C.L., Tanzi R.E. (2003). Copper, beta-amyloid, and Alzheimer’s disease: Tapping a sensitive connection. Proc. Natl. Acad. Sci. USA.

[71] Bush A.I., Curtain C.C. (2008). Twenty years of metallo-neurobiology: Where to now?. Eur. Biophys. J. EBJ.

[72] Jomova K., Vondrakova D., Lawson M., Valko M. (2010). Metals, oxidative stress and neurodegenerative disorders. Mol. Cell. Biochem..

[73] Monacelli F., Borghi R., Pacini D., Serrati C., Traverso N., Odetti P. (2014). Pentosidine determination in CSF: A potential biomarker of Alzheimer’s disease?. Clin. Chem. Lab. Med..

[74] Butterfield D.A., Reed T., Newman S.F., Sultana R. (2007). Roles of amyloid beta-peptide-associated oxidative stress and brain protein modifications in the pathogenesis of Alzheimer’s disease and mild cognitive impairment. Free Radic. Biol. Med..

[75] Polidori M.C., Mecocci P. (2002). Plasma susceptibility to free radical-induced antioxidant consumption and lipid peroxidation is increased in very old subjects with Alzheimer disease. J. Alzheimers Dis..

[76] Schipper H.M., Cisse S., Stopa E.G. (1995). Expression of heme oxygenase-1 in the senescent and Alzheimer-diseased brain. Ann. Neurol..

[77] Ischiropoulos H. (1998). Biological tyrosine nitration: A pathophysiological function of nitric oxide and reactive oxygen species. Arch. Biochem. Biophys..

[78] Garcia-Krauss A., Ferrada L., Astuya A., Salazar K., Cisternas P., Martinez F., Ramirez E., Nualart F. (2016). Dehydroascorbic acid promotes cell death in neurons under oxidative stress: A protective role for astrocytes. Mol. Neurobiol..

[79] May J.M. (2012). Vitamin c transport and its role in the central nervous system. Sub-Cell. Biochem..

[80] Sultana R., Mecocci P., Mangialasche F., Cecchetti R., Baglioni M., Butterfield D.A. (2011). Increased protein and lipid oxidative damage in mitochondria isolated from lymphocytes from patients with Alzheimer’s disease: Insights into the role of oxidative stress in Alzheimer’s disease and initial investigations into a potential biomarker for this dementing disorder. J. Alzheimers Dis..

[81] Dixit S., Bernardo A., Walker J.M., Kennard J.A., Kim G.Y., Kessler E.S., Harrison F.E. (2015). Vitamin C deficiency in the brain impairs cognition, increases amyloid accumulation and deposition, and oxidative stress in APP/PSEN1 and normally aging mice. ACS Chem. Neurosci..

[82] Parthasarathy S., Yoo B., McElheny D., Tay W., Ishii Y. (2014). Capturing a reactive state of amyloid aggregates: Nmr-based characterization of copper-bound Alzheimer disease amyloid beta-fibrils in a redox cycle. J. Biol. Chem..

[83] Berger T.M., Polidori M.C., Dabbagh A., Evans P.J., Halliwell B., Morrow J.D., Roberts L.J., Frei B. (1997). Antioxidant activity of vitamin C in iron-overloaded human plasma. J. Biol. Chem..

[84] Sil S., Ghosh T., Gupta P., Ghosh R., Kabir S.N., Roy A. (2016). Dual role of vitamin C on the neuroinflammation mediated neurodegeneration and memory impairments in colchicine induced rat model of Alzheimer disease. J. Mol. Neurosci. MN.

[85] Harrison F.E., Hosseini A.H., McDonald M.P., May J.M. (2009). Vitamin C reduces spatial learning deficits in middle-aged and very old APP/PSEN1 transgenic and wild-type mice. Pharmacol. Biochem. Behav..

[86] Huang J., May J.M. (2006). Ascorbic acid protects SH-SY5Y neuroblastoma cells from apoptosis and death induced by beta-amyloid. Brain Res..

[87] Ahmad A., Shah S.A., Badshah H., Kim M.J., Ali T., Yoon G.H., Kim T.H., Abid N.B., Rehman S.U., Khan S. (2016). Neuroprotection by vitamin c against ethanol-induced neuroinflammation associated neurodegeneration in the developing rat brain. CNS Neurol. Disord. Drug Targets.

[88] Ballaz S., Morales I., Rodriguez M., Obeso J.A. (2013). Ascorbate prevents cell death from prolonged exposure to glutamate in an in vitro model of human dopaminergic neurons. J. Neurosci. Res..

[89] Chan S., Kantham S., Rao V.M., Palanivelu M.K., Pham H.L., Shaw P.N., McGeary R.P., Ross B.P. (2016). Metal chelation, radical scavenging and inhibition of abeta(4)(2) fibrillation by food constituents in relation to Alzheimer’s disease. Food Chem..

[90] Polidori M.C., Pientka L., Mecocci P. (2012). A review of the major vascular risk factors related to Alzheimer’s disease. J. Alzheimers Dis..

[91] Murakami K., Murata N., Ozawa Y., Kinoshita N., Irie K., Shirasawa T., Shimizu T. (2011). Vitamin c restores behavioral deficits and amyloid-beta oligomerization without affecting plaque formation in a mouse model of Alzheimer’s disease. J. Alzheimers Dis..

[92] Hauss-Wegrzyniak B., Wenk G.L. (2002). Beta-amyloid deposition in the brains of rats chronically infused with thiorphan or lipopolysaccharide: The role of ascorbic acid in the vehicle. Neurosci. Lett..

[93] Deane R., Bell R.D., Sagare A., Zlokovic B.V. (2009). Clearance of amyloid-beta peptide across the blood-brain barrier: Implication for therapies in Alzheimer’s disease. CNS Neurol. Disord. Drug Targets.

[94] Dede D.S., Yavuz B., Yavuz B.B., Cankurtaran M., Halil M., Ulger Z., Cankurtaran E.S., Aytemir K., Kabakci G., Ariogul S. (2007). Assessment of endothelial function in Alzheimer’s disease: Is Alzheimer’s disease a vascular disease?. J. Am. Geriatr. Soc..

[95] Lam V., Hackett M., Takechi R. (2016). Antioxidants and dementia risk: Consideration through a cerebrovascular perspective. Nutrients.

[96] Sarkar S., Mukherjee A., Swarnakar S., Das N. (2016). Nanocapsulated ascorbic acid in combating cerebral ischemia reperfusion-induced oxidative injury in rat brain. Curr. Alzheimer Res..

[97] Dringen R., Kussmaul L., Gutterer J.M., Hirrlinger J., Hamprecht B. (1999). The glutathione system of peroxide detoxification is less efficient in neurons than in astroglial cells. J. Neurochem..

[98] Rohl C., Armbrust E., Herbst E., Jess A., Gulden M., Maser E., Rimbach G., Bosch-Saadatmandi C. (2010). Mechanisms involved in the modulation of astroglial resistance to oxidative stress induced by activated microglia: Antioxidative systems, peroxide elimination, radical generation, lipid peroxidation. Neurotox. Res..

[99] Heo J.H., Hyon L., Lee K.M. (2013). The possible role of antioxidant vitamin C in Alzheimer’s disease treatment and prevention. Am. J. Alzheimers Dis. Other Dement..

[100] Rosales-Corral S., Tan D.X., Reiter R.J., Valdivia-Velazquez M., Martinez-Barboza G., Acosta-Martinez J.P., Ortiz G.G. (2003). Orally administered melatonin reduces oxidative stress and proinflammatory cytokines induced by amyloid-beta peptide in rat brain: A comparative, in vivo study versus vitamin C and E. J. Pineal Res..

[101] Dhingra D., Parle M., Kulkarni S.K. (2006). Comparative brain cholinesterase-inhibiting activity of *Glycyrrhiza glabra*, *Myristica fragrans*, ascorbic acid, and metrifonate in mice. J. Med. Food.

[102] Kuo C.H., Hata F., Yoshida H., Yamatodani A., Wada H. (1979). Effect of ascorbic acid on release of acetylcholine from synaptic vesicles prepared from different species of animals and release of noradrenaline from synaptic vesicles of rat brain. Life Sci..

[103] Cheng F., Cappai R., Ciccotosto G.D., Svensson G., Multhaup G., Fransson L.A., Mani K. (2011). Suppression of amyloid beta a11 antibody immunoreactivity by vitamin C: Possible role of heparan sulfate oligosaccharides derived from glypican-1 by ascorbate-induced, nitric oxide (no)-catalyzed degradation. J. Biol. Chem..

[104] Jiang D., Li X., Liu L., Yagnik G.B., Zhou F. (2010). Reaction rates and mechanism of the ascorbic acid oxidation by molecular oxygen facilitated by cu(II)-containing amyloid-beta complexes and aggregates. J. Phys. Chem. B.

[105] Chambial S., Dwivedi S., Shukla K.K., John P.J., Sharma P. (2013). Vitamin c in disease prevention and cure: An overview. Indian J. Clin. Biochem. IJCB.

[106] Rahal A., Kumar A., Singh V., Yadav B., Tiwari R., Chakraborty S., Dhama K. (2014). Oxidative stress, prooxidants, and antioxidants: The interplay. BioMed Res. Int..

[107] Cheng R., Lin B., Lee K.W., Ortwerth B.J. (2001). Similarity of the yellow chromophores isolated from human cataracts with those from ascorbic acid-modified calf lens proteins: Evidence for ascorbic acid glycation during cataract formation. Biochim. Biophys. Acta.

[108] Carr A., Frei B. (1999). Does vitamin c act as a pro-oxidant under physiological conditions?. FASEB J. Off. Publ. Fed. Am. Soc. Exp. Biol..

[109] Valko M., Morris H., Cronin M.T. (2005). Metals, toxicity and oxidative stress. Curr. Med. Chem..

[110] Dikalov S.I., Vitek M.P., Mason R.P. (2004). Cupric-amyloid beta peptide complex stimulates oxidation of ascorbate and generation of hydroxyl radical. Free Radic. Biol. Med..

[111] Shearer J., Szalai V.A. (2008). The amyloid-beta peptide of Alzheimer’s disease binds Cu(I) in a linear Bis-His coordination environment: Insight into a possible neuroprotective mechanism for the amyloid-beta peptide. J. Am. Chem. Soc..

[112] Nadal R.C., Rigby S.E., Viles J.H. (2008). Amyloid beta-Cu^2+^ complexes in both monomeric and fibrillar forms do not generate H_2_O_2_ catalytically but quench hydroxyl radicals. Biochemistry.

[113] Huang Y.N., Lai C.C., Chiu C.T., Lin J.J., Wang J.Y. (2014). l-ascorbate attenuates the endotoxin-induced production of inflammatory mediators by inhibiting MAPK activation and NF-kappaB translocation in cortical neurons/glia Cocultures. PloS ONE.

[114] Kook S.Y., Lee K.M., Kim Y., Cha M.Y., Kang S., Baik S.H., Lee H., Park R., Mook-Jung I. (2014). High-dose of vitamin c supplementation reduces amyloid plaque burden and ameliorates pathological changes in the brain of 5XFAD mice. Cell Death Dis..

[115] Polidori M.C., Ruggiero C., Croce M.F., Raichi T., Mangialasche F., Cecchetti R., Pelini L., Paolacci L., Ercolani S., Mecocci P. (2015). Association of increased carotid intima-media thickness and lower plasma levels of vitamin C and vitamin E in old age subjects: Implications for Alzheimer’s disease. J. Neural Transm..

[116] Bomboi G., Castello L., Cosentino F., Giubilei F., Orzi F., Volpe M. (2010). Alzheimer’s disease and endothelial dysfunction. Neurol. Sci. Off. J. Ital. Neurol. Soc. Ital. Soc. Clin. Neurophysiol..

[117] May J.M., Qu Z.C. (2010). Ascorbic acid prevents increased endothelial permeability caused by oxidized low density lipoprotein. Free Radic. Res..

[118] May J.M., Harrison F.E. (2013). Role of vitamin C in the function of the vascular endothelium. Antioxid. Redox Signal..

[119] Polidori M.C., Pientka L. (2012). Bridging the pathophysiology of Alzheimer’s disease with vascular pathology: The feed-back, the feed-forward, and oxidative stress. J. Alzheimers Dis..

[120] Miller M.C., Tavares R., Johanson C.E., Hovanesian V., Donahue J.E., Gonzalez L., Silverberg G.D., Stopa E.G. (2008). Hippocampal rage immunoreactivity in early and advanced Alzheimer’s disease. Brain Res..

[121] Lin J.L., Huang Y.H., Shen Y.C., Huang H.C., Liu P.H. (2010). Ascorbic acid prevents blood-brain barrier disruption and sensory deficit caused by sustained compression of primary somatosensory cortex. J. Cereb. Blood Flow Metab. Off. J. Int. Soc. Cereb. Blood Flow Metab..

[122] May J.M., Qu Z.C. (2011). Nitric oxide mediates tightening of the endothelial barrier by ascorbic acid. Biochem. Biophys. Res. Commun..

[123] Scuteri A., Tesauro M., Appolloni S., Preziosi F., Brancati A.M., Volpe M. (2007). Arterial stiffness as an independent predictor of longitudinal changes in cognitive function in the older individual. J. Hypertens..

[124] Ellingsen I., Seljeflot I., Arnesen H., Tonstad S. (2009). Vitamin C consumption is associated with less progression in carotid intima media thickness in elderly men: A 3-year intervention study. Nutr. Metab. Cardiovasc. Dis. NMCD.

[125] Donahue J.E., Flaherty S.L., Johanson C.E., Duncan J.A., Silverberg G.D., Miller M.C., Tavares R., Yang W., Wu Q., Sabo E. (2006). Rage, lrp-1, and amyloid-beta protein in Alzheimer’s disease. Acta Neuropathol..

[126] Pleiner J., Schaller G., Mittermayer F., Marsik C., MacAllister R.J., Kapiotis S., Ziegler S., Ferlitsch A., Wolzt M. (2008). Intra-arterial vitamin C prevents endothelial dysfunction caused by ischemia-reperfusion. Atherosclerosis.

[127] Scorza G., Pietraforte D., Minetti M. (1997). Role of ascorbate and protein thiols in the release of nitric oxide from *S*-nitroso-albumin and *S*-nitroso-glutathione in human plasma. Free Radic. Biol. Med..

[128] Allahtavakoli M., Amin F., Esmaeeli-Nadimi A., Shamsizadeh A., Kazemi-Arababadi M., Kennedy D. (2015). Ascorbic acid reduces the adverse effects of delayed administration of tissue plasminogen activator in a rat stroke model. Basic Clin. Pharmacol. Toxicol..

[129] Song J., Park J., Kim J.H., Choi J.Y., Kim J.Y., Lee K.M., Lee J.E. (2015). Dehydroascorbic acid attenuates ischemic brain edema and neurotoxicity in cerebral ischemia: An in vivo study. Exp. Neurobiol..

[130] Allen C.L., Bayraktutan U. (2009). Antioxidants attenuate hyperglycaemia-mediated brain endothelial cell dysfunction and blood-brain barrier hyperpermeability. Diabetes Obes. Metab..

[131] Bowman G.L. (2012). Ascorbic acid, cognitive function, and Alzheimer’s disease: A current review and future direction. BioFactors.

[132] Bowman G.L., Dodge H., Frei B., Calabrese C., Oken B.S., Kaye J.A., Quinn J.F. (2009). Ascorbic acid and rates of cognitive decline in Alzheimer’s disease. J. Alzheimers Dis..

[133] Bowman G.L., Kaye J.A., Moore M., Waichunas D., Carlson N.E., Quinn J.F. (2007). Blood-brain barrier impairment in alzheimer disease: Stability and functional significance. Neurology.

[134] Spector R., Johanson C.E. (2013). Sustained choroid plexus function in human elderly and Alzheimer’s disease patients. Fluids Barriers CNS.

[135] Charlton K.E., Rabinowitz T.L., Geffen L.N., Dhansay M.A. (2004). Lowered plasma vitamin C, but not vitamin E, concentrations in dementia patients. J. Nutr. Health Aging.

[136] Rinaldi P., Polidori M.C., Metastasio A., Mariani E., Mattioli P., Cherubini A., Catani M., Cecchetti R., Senin U., Mecocci P. (2003). Plasma antioxidants are similarly depleted in mild cognitive impairment and in Alzheimer’s disease. Neurobiol. Aging.

[137] Morris M.C., Beckett L.A., Scherr P.A., Hebert L.E., Bennett D.A., Field T.S., Evans D.A. (1998). Vitamin E and vitamin C supplement use and risk of incident Alzheimer disease. Alzheimer Dis. Assoc. Disord..

[138] Zandi P.P., Anthony J.C., Khachaturian A.S., Stone S.V., Gustafson D., Tschanz J.T., Norton M.C., Welsh-Bohmer K.A., Breitner J.C., Cache County Study G. (2004). Reduced risk of Alzheimer disease in users of antioxidant vitamin supplements: The cache county study. Arch. Neurol..

[139] Devore E.E., Kang J.H., Stampfer M.J., Grodstein F. (2013). The association of antioxidants and cognition in the nurses’ health study. Am. J. Epidemiol..

[140] Engelhart M.J., Geerlings M.I., Ruitenberg A., van Swieten J.C., Hofman A., Witteman J.C., Breteler M.M. (2002). Dietary intake of antioxidants and risk of Alzheimer disease. JAMA.

[141] Quinn J., Suh J., Moore M.M., Kaye J., Frei B. (2003). Antioxidants in Alzheimer’s disease-vitamin c delivery to a demanding brain. J. Alzheimers Dis..

[142] Fillenbaum G.G., Kuchibhatla M.N., Hanlon J.T., Artz M.B., Pieper C.F., Schmader K.E., Dysken M.W., Gray S.L. (2005). Dementia and Alzheimer’s disease in community-dwelling elders taking vitamin C and/or vitamin E. Ann. Pharmacother..

[143] Harrison F.E. (2012). A critical review of vitamin C for the prevention of age-related cognitive decline and Alzheimer’s disease. J. Alzheimers Dis..

[144] Emir U.E., Raatz S., McPherson S., Hodges J.S., Torkelson C., Tawfik P., White T., Terpstra M. (2011). Noninvasive quantification of ascorbate and glutathione concentration in the elderly human brain. NMR Biomed..

[145] Minor E.A., Court B.L., Young J.I., Wang G. (2013). Ascorbate induces ten-eleven translocation (Tet) methylcytosine dioxygenase-mediated generation of 5-hydroxymethylcytosine. J. Biol. Chem..

[146] Smuda M., Glomb M.A. (2013). Maillard degradation pathways of vitamin C. Angew. Chem..

